# The use of a silicone-coated acrylic vaginal stent in McIndoe vaginoplasty and review of the literature concerning silicone-based vaginal stents: a case report

**DOI:** 10.1186/1471-2482-7-13

**Published:** 2007-07-10

**Authors:** Ayhan Coskun, Yusuf Kenan Coban, Mehmet Ali Vardar, Ahmet Cemil Dalay

**Affiliations:** 1 Department of Obstetrics and Gynecology, Sutcu Imam University School of Medicine, Kahramanmaras, Turkey; 2 Department of Plastic Surgery, Sutcu Imam University School of Medicine, Kahramanmaras, Turkey; 3 Department of Obstetrics and Gynecology, Cukurova University School of Medicine, Adana, Turkey; 4 Department of Plastic Surgery, Cukurova University School of Medicine, Adana, Turkey

## Abstract

**Background:**

Mc Indoe vaginoplasty is one of the mostly performed surgical interventions in Mullerian agenesis.

**Case presentations:**

We present our experience on the use of a new designed vaginal stent that was coated with silicone in two mullerian agenesis cases who had Mc Indoe vaginoplasty. Both full thickness and splitt thickness skin graft were used with the stent. No graft loss or hyperthrophic scarring which may be seen at the apex of neovagina after Mc Indoe vaginoplasty was observed during the follow-up period and adequate neovaginal depth were obtained in both of the patients.

**Conclusion:**

We think that the incorporation of silicone to a vaginal stent for postoperative wound care improves skin graft take and decreases a possible constriction band formation in neovagina.

## Background

The correction of vaginal agenesis requires the creation of a neovaginal cavity that is dissected between the bladder and the rectum [1]. The technique needs to use the split-thickness skin graft or full-thickness skin graft. The procedure is not entirely satisfactory in cases of reconstructed vaginal stenosis, inadequate vaginal length. In order to prevent a possible contraction of the reconstructed vagina, a long-term vaginal stent use is required to maintain vaginal width and depth. The vacuum assisted closure-system (VAC) has recently been introduced to improve the take of skin graft in vaginal reconstruction and it has been reported to exclude the need for vaginal stent [2]. Although new techniques which does not necessitate a prolonged dilatation is developed, McIndoe's method is still one of the very popular methods of vaginoplasty and it has been shown to be effective in creation of a neovagina for patients with mullerian agenesis [3]. Several vaginal stents have been described for postoperative maintanence after Mc Indoe vaginoplasty [4-9]. We present our experience with a new silicone coated acrylic stent in 2 vaginoplasty patients.

### The silicone coated vaginal stent

**Figure 1:**
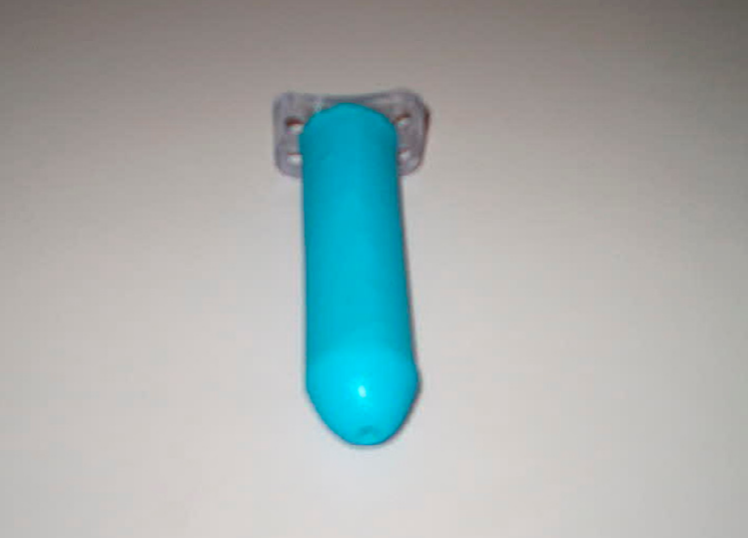
The silicone-coated vaginal stent is seen.

Silicone is the most widely used biomaterial in plastic surgery field for different purposes due to its high biocompatibilty. We incorporated silicone to a new designed vaginal stent. The idea has been originated from the fact that using silicone gel sheeting is an effective method for skin graft stabilization [10]. The stent is 3 cm in diameter and 11–12 cm in lenght. It's inner body is composed of rigid acrylic mold and the outer surface is coated with solid silicone. One hole at the distal end of the stent for drainage at the early postoperative period and four holes at the proximal plastic plate for securing the stent to body are designed (figure 1).

### Stent preparation

The stent has three components which are formed by inner acrylic (akrileks, Turkey), outer silicone (monopren liquid siliconei silimed, Germany) and a perpendicularlary attached plastic with holes for fixation. The main body is consisted of acrylic solid material which is 12,5 cm long. Its diameter is 3 cm and clyndric construction resembles a 20 cc syrnge. The plastic plate that was attached to the main body was consisted of a material which is used for dental mold applications (meraport, germany). These three components are prepared sparately and then they are combined to form an compact unit under 80–90°C for 5 minutes. The stent is sterilized under etilen oxide for surgical usage. The holes of plastic component is designed for fixation of the stent to the body. The liquid silicone is applicated to the outer surface of acrylic stent just before the heating process which converts it to a solid silicone

### Surgical Technique

The vaginal agenesia is opened through an I incision and then two sides of the central fibrotic band are dissected. After having hemostasis and pouches at 8 cm long bilaterally, the central band is excised. The newly created vaginal pouche is rechecked for hemostasis and then the stent is inserted to the pouche for checking of stent compliance with the neovagina. At this time, the harvested skin graft (full-thickness or splitt-thickness) is wrapped around the silicone coated stent with an antibiotic oinment (furaderm, toprak ilaç, turkey). The edges of skin graft is sutured to each other with a resorbable suture material. The application of the skin graft to the wound necessiates a great care. During the graft insertion, an or two assistants make a wide exposure to vaginal pouche with ecartors and the stent with the skin graft is settled gently. From this time of operation every movement must be done carefully avoiding graft distorsion or tear. The distal edges of skin graft can be sutured to the edges of openning incision at this time. The stent is fixated to a belt around the body with inserting sterile serum set through the holes. The most important point in the stent fixation is to hold a parellel fixation to normal vagen axis, otherwise meatal or urethral necrosis may occur due to undue pressure resulted from improper stent fixation. This paralel fixation prevents the stent from prolapsing at the early postoperative period. The incorporation of silicone has the advantage of a superior skin graft take with relieving a possible pressure necrosis around the stent.

## Case presentations

### Case 1

**Figure 2:**
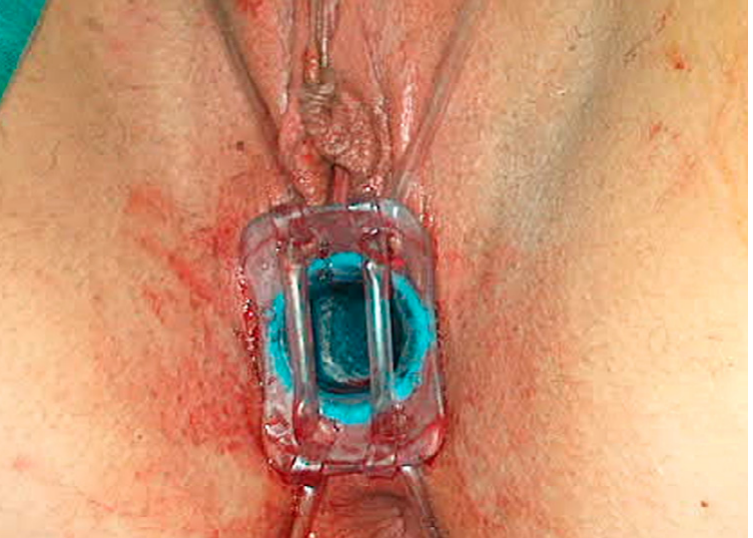
The stent is seen after securing to body with intravenous infusion set tubes which tied to belt in the patient.

**Figure 3:**
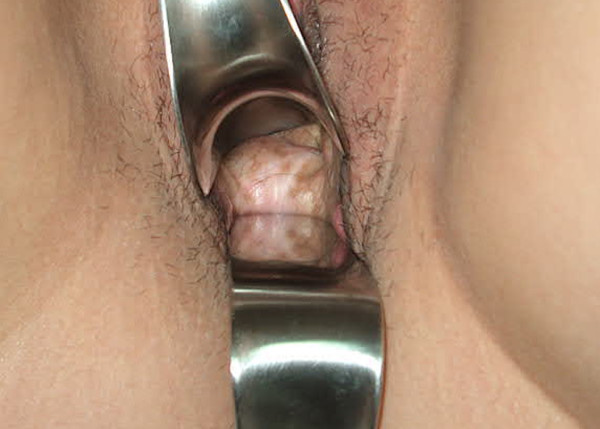
1 year later the view of neovagina of the case 1.

21 years old female was referred as complete vaginal agenesis. She was examined and Rokitansky Kustner was diagnosed. She was operated under general anesthesia in october 2004. Full-thickness skin graft taken from inguinal region was used. The prosthesis was secured in place for 5 days after the operation and then it was gently removed for first look to the grafts. For the first postoperative three weeks, wound care was done with serum physiologic, furacine (nitrofurantione) cream and rifocine (rifampisine). Then the prosthesis kept in place for 7 months with cleansing it for one time in every week. The only problem encountered during postoperative course, a granulation tissue formation along with the graft suture lines and electrocautery was applicated for 2 times. Additionaly aloe-vera cream was used for wound care during the period of 2nd–7th postoperative months in which a complete epithelization was achieved. She has been sexually active without any problem (Figure 2 and 3). Hair growth problem in the newly created vaginal space was solved with an epilatory cream application.

### Case 2

**Figure 4:**
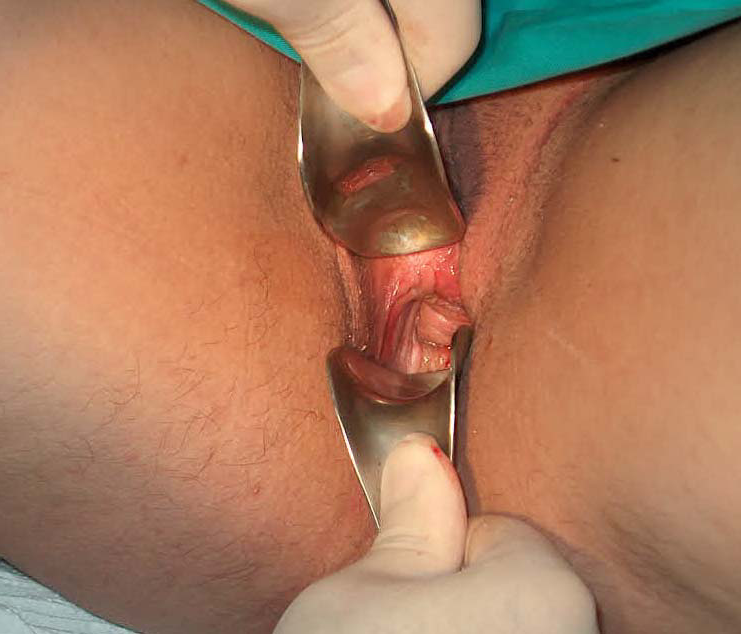
3 months after Mc Indoe vaginoplasty, the neovagina in the case 2 is seen.

30 year-old female was also referred for amenore. Gynecologic examination showed that vaginal agenesis was present and a rudimentary uterus was palpated with rectal examination. Ovaries were seen normal dimentions by ultrasounographic evaluation. A further Caryotype analysis showed 46XX and Mayer-Rokitansy-Küster-Hauser syndrome (mullerian agenesis) was diagnosed for the patient. She was operated at january of 2006 with the same technique. A split-thickness skin graft was used and a monoblock skin-graft was wrapped around the prosthesis and placed into the pouche which was dissected according to Mc Indoe thechnique. 4/0 chrome catgut (Dogsan, Turkey)was used for suturing the edges of grafts. The same wound care protocol was applied to the patient and no skin graft loss was seen. She was followed up for 5 months no complication was seen except a minimal granulation tissue formation. This was also managed with electrocautery. An vaginal depth of 11 × 3 cm was obtained and maintained in the patient. The patient is still under follow-up at early postoperative period and an ideal wound healing course was also noted (Figure 4).

## Comments

We executed two vaginoplasty using the stent. Both full thickness skin graft and splitt thickness skin graft were used with the stent without any complication. Securing the skin grafts to the stent is easy for surgeon as skin grafts easily adopted to the stent. No graft loss was seen in our experience. One patient was followed up for more than one year and the other was for 7 months. No vaginal constriction band was observed in these patients and a neovaginal depth of 3 × 9 cm were obtained and maintained in the cases. We think that the use of a silicone incorporated stent with Mc Indoee vaginoplasty has double advantages. The first is that it has a superior skin graft intake and the second advantage of it's use in Mc indoe vaginoplasty is that it prevents a possible hypertophic scar formation after epitheliazation completed.

The creation of vagina that has a satisfactory appearance function and feeling as the aim of vaginoplasty should always be considered [11]. In a retrospective study 75% of Mc indoe vaginoplasty patients stated that the procedure improved their quality of life [12]. If surgery is required for creating a functionally useful vagina the primary operation should be definite and performed by well-trained experts [13]. Some wound contraction may occur at apex of neovaginoplasty after skin graft vaginoplasty techniques [3]. The patient wears the stent day and night for 3–4 months and the use a silicone coated stent during remodeling phase of a grafted vaginoplasty case may help to reduce such a wound contraction in neovagina. So the stent is not only used at peroperative period after sterilization, but also used for postoperative period for stenting. We recommend to use this kind of silicone coated vaginal stent in the postoperative management of Mc Indoe vaginoplasty patients.

## Competing interests

The author(s) declare that they have no competing interests.

## Authors' contributions

All the authors have been involved in literature search, writing and final reviewing of this manuscript.

## Pre-publication history

The pre-publication history for this paper can be accessed here:


